# The effect of exposure to fiction on attributional complexity, egocentric bias and accuracy in social perception

**DOI:** 10.1371/journal.pone.0233378

**Published:** 2020-05-29

**Authors:** Emanuele Castano, Alison Jane Martingano, Pietro Perconti

**Affiliations:** 1 Department of Psychology and Cognitive Science, University of Trento, Rovereto, Italy; 2 Institute for Biomedical Research and Innovation, National Research Council, Palermo, Italy; 3 Department of Political Science and International Relations, Sarajevo School of Science and Technology, Sarajevo, Bosnia-Herzegovina; 4 Department of Psychology, The New School for Social Research, New York, NY, United States of America; 5 Department of Cognitive Science, University of Messina, Messina, Italy; Educational Testing Service, UNITED STATES

## Abstract

We investigated the effects of long-term exposure to literary and popular fiction on attributional complexity, egocentric bias and accuracy. Results of a pre-registered study showed that exposure to literary fiction is positively associated with scores on the attributional complexity scale. Literary fiction is also associated with accuracy in mentalizing, measured via the Reading the Mind in the Eyes test, and with accuracy in predicting average social attitudes. The predicted negative association between literary fiction and egocentric bias emerged only when education and gender were controlled for–a covariance analysis that was not pre-registered. Exposure to popular fiction is associated solely with attributional complexity, but negatively. We discuss the significance of these findings in the context of the emerging literature regarding the relationship between fiction and social cognition.

## Introduction

As children we listen to (and fabricate) stories all day long, and as adults we end the day by reading, watching, and increasingly by playing, stories. We love stories because they are entertaining, they teach us about the world we live in and, just as social interaction, help us to build the cognitive processes needed to learn about the world [1]. Scholarly work in evolutionary theory and anthropology suggests that stories played a significant role in the evolution of human cognition [2–4]. Over the past decade research has investigated the processes involved in the mental construction of fictional worlds [5], how readers are transported into such worlds [6], and the impact which engagement with fiction has on cognition [7]. Particular attention has been paid to the relationship between engagement with fiction and mentalizing processes from the perspective of cognitive science (for a review, [8]) and in the emerging field of cognitive literary theory (e.g., [9]).

In this article, we focus on how two types of fiction, literary and popular, differentially impact not only mentalizing but also other social cognition processes. After briefly reviewing the characteristics of popular and literary fiction, we present a rationale and outline a series of hypotheses with regard to the impact of these two types of fiction on attributional complexity, ego-centric bias, and accuracy in social perception, and present results of a pre-registered study testing such hypotheses.

### Literary and popular fiction

This distinction between popular and literary fiction is recognized by publishers, who list literary fiction as a separate publishing category, as well as by critics who award different prizes for popular (e.g., Prix Paul-Féval, IBPA Benjamin Franklin Award) *versus* literary fiction (e.g., Grand prix du roman de l'Académie française; Premio Strega, National Book Award). Popular and literary fiction are also thought to attract readers for different reasons—entertainment and escape for popular fiction, understanding and engagement for literary fiction [10–12]. The ecological validity of the distinction is matched by scholarly work which discusses the complexity and indeterminacy of the plots and characters of literary, as compared to those of popular, fiction (e.g., [13]).

Bruner [14] proposed that literary fiction engages readers in a discourse that forces them to fill in narrative gaps and to search for meanings "among a spectrum of possible meanings” ([14], p. 25). According to Bruner, literary fiction triggers presupposition (a focus on implicit meanings), subjectification [depicting reality “through the filter of the consciousness of protagonists in the story” ([14], p. 25), and the adoption of multiple perspectives (perceiving the world simultaneously from different view- points). This characterization of literary fiction builds on Barthes’ [15] definition of *writerly* fiction as distinguished from *readerly* fiction.

In *readerly* (popular) fiction, readers have a more passive role; they accept meanings that are provided to them “prêt-à-porter.” Characters are “types”: consistent, predictable, and rather unidimensional. They may surprise us, but when they do so, it is because they switch from one type to another. Readerly fiction does not obey by the “show don’t tell” rule [16]; it tells. On the other hand, *writerly* (literary) fiction requires a much more active role from the readers who are not told but (merely) shown, and as a consequence have to extract or even construct meaning for themselves. Literary texts traffic “in human possibilities rather than in settled certainties” ([14], p. 26).

A similar point is made by Bakhtin [17] according to whom literary fiction lacks the unique authorial voice usually found in popular fiction and thus a unique truth about the story and its characters. Instead, literary fiction invites the readers to contribute their own perspectives. Bakhtin refers to it as “polyphonic,” in as much it elicits a diversity of simultaneous points of view and voices, each drawing its own inferences and constructing its own meaning.

Meaning inference and construction does not occur uniquely when individuals read literary fiction. Linguistic meaning is matter of pragmatic enrichment; there is a gap between what is literally said and the speaker’s intention, and this gap is filled by drawing inferences, guided by pragmatic competence [18, 19]. Research and theorizing on narrative text comprehension have long discussed the inferential processes that readers engage in when interacting with narratives—the “search (or effort) after meaning” [20]. In this scholarly literature, several accounts have been proposed which vary, for instance, in the types of online inferences that readers make; ranging from referential, to causal, to inference about the author’s intent. To discuss these theories is beyond the scope of this article (for a review, see [21]), but we agree that readers routinely engage in inferential processes at various levels when reading all fiction. Our claim here is narrower, it concerns the differences between literary and popular fiction in terms of what is required by, and elicited in, readers, as they navigate fictional worlds and their characters.

We have seen above that compared to popular fiction, the obscure and complex world depicted in literary fiction forces the reader to dwell in uncertainty, to navigate possibilities, and to consider multiple perspectives [14, 17]. Another difference between popular and literary fiction is in their relative emphasis on the plot *versus* characters. Popular fiction is more concerned with the plot; its characters provide clear anchors around which the plot can be developed [22]. Literary fiction is more concerned with the inner life of the characters; it is concerned with the idiosyncrasy and uniqueness [11] and, possibly the ultimate inaccessibility of human consciousness [23].

A consequence of this emphasis on inner life is that literary fiction highlights the subjective over the objective, uncertainty and multiplicity over certainty and singularity. Another, related consequence is that readers are invited to pay greater attention to the workings of the mind. While all fiction requires understanding characters’ embedded mental states, literary fiction “make[s] the reader infer implied mental states in addition to (and sometimes instead of) spelling some out” ([24], p. 5). Consistent with this rationale, compared to their popular counterparts, literary characters are perceived by readers as more complex [25].

### From fiction to social cognition

We have argued that one dimension which differentiates literary and popular fiction is *complexity;* literary fiction paints a more complex picture of human affairs, and of the human psyche, than popular fiction. If that is the case, and fiction is indeed a gym for social cognition processes that are the same as those utilized outside of the fictional context, we should find that readers of literary fiction develop more complex schemas about others, their behavior, and about the social world they inhabit. In the social domain, an attempt to capture the complexity of thinking is represented by the construct of Attributional Complexity [26]. The construct was proposed as an integration of several perspectives on cognitive complexity and includes the motivation to understand human behavior, along with the preference for complex explanations of it. The construct further includes the presence of meta-cognitive elements in the process through which explanations are reached, and the tendency to infer abstract attributions, both for internal and external factors. This presupposes the awareness that behavior is affected by external forces and by interaction with others. The awareness that not immediately-present factors (sometime stemming from a distal past) may also affect behavior, is an additional dimension of the concept of attributional complexity. We hypothesize that exposure to literary, but not to popular fiction, should predict greater attributional complexity.

We argued earlier that literary and popular fiction differ in the degree to which they encourage perspective-taking. Compared to popular fiction, literary fiction is theorized to encourage the simultaneous entertaining of a multiplicity of perspectives [14, 17]. To take into consideration other perspectives should reduce the tendency to see our own construals as objective, and thus to rely less on them when making attributions about others and predicting their behavior [27]. Exposure to literary fiction, therefore, should lead to a reduction of ego-centric biases, such as the false-consensus effect: the tendency to overestimate the extent to which others think the same way we do, and hold the same beliefs, attitudes, etc. [28]. We therefore hypothesize that exposure to literary, but not popular fiction, should negatively predict false-consensus.

If a tendency to consider and appreciate other perspectives reduces ego-centric biases it should therefore enhance *accuracy*, which we define as the capacity to accurately represent others’ opinion, beliefs, intentions, emotion or attitudes at the individual mind level, or at the social level.

At the mind level, accuracy is typically measured via the Theory of Mind tests, performance on which research has demonstrated to be boosted by exposure to literary fiction [25, 29–35]. At the social level, an indicator of social accuracy can be obtained through the same paradigm used to investigate false-consensus effects—by looking at the correlation between observed and estimated popularities [36]. We hypothesize that exposure to literary, but not popular fiction, should predict greater mind accuracy and social accuracy. To test the hypotheses outlined above we conducted a pre-registered study. (https://aspredicted.org/m5ja6.pdf).

## Method

Unless otherwise specified, the description of the methods and statistics described below (including number of participants, exclusion criteria, inferential statistics used) were as pre-registered. Other variables measured in this data collection, but not reported here, are: Charitable Donations (assessed via the presentation of different scenarios of persons in need), the Moral Foundation Questionnaire and the Moral Judgment Task. A target sample of 500 participants were pre-registered, primarily because we intended to have at least 15 respondents per item to conduct a factor analysis on the attributional complexity scales (28 items). Results of this factor analysis, registered as exploratory, are not reported here.

### Participants

Participants (502) were recruited via Amazon Mechanical Turk (MTurk) and paid $5 for their participation. MTurk is a crowdsourcing marketplace for work that is used extensively in behavioral research and has been proven to be a reliable source of good quality data [37]. Participants were excluded for not completing the study (N = 1), for selecting more foils (i.e. names of non-existing authors) than authors (N = 3) or selecting no authors at all (N = 2) on the Author Recognition Test, and for performing at below-chance levels on the Reading the Mind in the Eyes Test (N = 4). These exclusions were pre-registered and in line with previous research. The meaning of these variable-specific exclusions will become clearer in light of the descriptions of the Author Recognition Test and the Reading the Mind in the Eyes Test, provided below. The final sample comprised 493 participants (63% female; 0.4% undeclared; age *M* = 34.8, *SD* = 11.56). Participants also indicated their level of education on a scale 1–5 corresponding to “some high school” (.61%), “high school” (10.55%), “some college” (36.11%), “college graduate” (35.29%), and “postgraduate” (17.44%), and their native language (97.15% indicated English). This project was approved by The New School’s Human Research Protection Program.

### Measures

#### Exposure to fiction

Long-term exposure to literary and popular fiction was measured via a modified version of the Author Recognition Test (ART) [38]. The ART displays an extensive list of names to participants and asks them to identify any authors they recognize. While recognition of authors’ names is not a direct measure of engagement with fiction, and it can be influenced by other factors besides fiction reading, the ART is considered preferable to other self-report measures that are more likely to be influenced by social desirability concerns. In the ART, the presence of an equal number of non-authors and fiction authors helps to discourage guessing, making this form of measurement less likely to be influenced by a desire to present oneself as a more avid reader than is truly the case [39]. Overall, the ART has been shown to predict participants’ actual engagement with fiction [39–41].

Modified versions of the ART have been used to distinguish rates of reading nonfiction and fiction (e.g., [42, 43]), as well as different genres of fiction (e.g., [44, 45]. As in previous studies ([25, 31]; see also [46]), here we computed two main scores based on participants’ answers to the ART: proportions of correctly identified literary authors (Literary: *M* = .29; *SD* = .22) and popular authors (Popular: *M* = .28; *SD* = .21). A third score, Foils, was also computed by calculating the proportions of non-authors that were selected, to be used as covariate.

#### Attributional complexity

The Attributional Complexity scale was used (ACS; [26]). The ACS comprises of 28-item scale measuring seven dimensions of attribution that are typically combined into one single factor displaying adequate internal and test-retest reliability. A composite score was computed by averaging all items (Cronbach’s alpha = .91; *M* = 5.22; *SD* = .81).

#### Egocentric bias

A false consensus paradigm was used in which participants are presented with 20 statements taken from the Minnesota Multiphasic Personality Index and asked: “For each item, please indicate whether YOU agree or disagree with that item” and “For each item, please estimate the percentage (0–100%) of other people who would agree with that item.” [47]. Examples of items are “I like to read newspaper articles on crime,” “I enjoy the excitement of a crowd.” To measure bias, a True False Consensus score (TFC) was obtained by computing the correlation of personal endorsement and the difference between estimated and actual endorsement of others [48]. Although not specified in the pre-registration, we also computed and analyzed a Partial True False Consensus score (P-TFC; correlation between endorsement and estimate popularity, controlling for true popularity as obtained in the sample). It has been suggested that the P-TFC provides a better measure of the egocentric bias [36]. Both measures were transformed to z-scores [48]. (TFC; *M* = .03; *SD* = .33; P-TFC; *M* = .29; *SD* = .36).

#### Accuracy

Mind accuracy was assessed via the Reading the Mind in the Eyes Test (RMET; [49]). The RMET contains 36 trials in which participants are shown an image of an actor’s eyes and are tasked with selecting the mental state which matches the expression by choosing one of four alternatives. Scores are computed by summing the number of correct answers (Mind Accuracy: *M* = 26.12; *SD* = 4.35). Social accuracy was assessed by computing the correlation between estimated popularity and true popularity [36], obtained via the same task used to measure ego-centric bias and described above (Social Accuracy; *M* = .59; *SD* = .24). The higher the score, the greater the accuracy.

## Results

A multiple regression approach was used, in which Literary and Popular ART scores were included as predictors, and attributional complexity, egocentric bias and accuracy measures as criteria. Foils (from the ART), was also included as covariate. Data analyses were performed using SAS (SAS Studio 3.8, Sas Institute Inc., Cary, NC). See Table 1 for results. Exposure to Literary and Popular fiction positively and negatively predicted attributional complexity respectively. For both egocentric bias measures (TFC and PCTF), Literary was a negative, but only marginal predictor, while Popular was not a predictor of either measures. For accuracy measures, exposure to Literary fiction positively predicted both Mind Accuracy and Social Accuracy, while exposure to Popular fiction did not predict either. Table 2 reports correlations between the criterion variables used in the analyses above as well as education.

**Table 1:** Results of multiple regression conducted separately for each DV, using literary and popular ART scores as predictors, controlling for ART foils.

Criterion	N	Predictors	*b*	95% CI		*SE*	*β*	*t*	*p*
Attributional Complexity	493	Literary	1.38	0.89	1.87	0.25	0.37	5.52	< .001
		Popular	-0.66	-1.17	-0.16	0.26	-0.17	-2.58	0.01
Egocentric Bias-TFC	491	Literary	-0.18	-0.38	0.03	0.11	-0.11	-1.68	0.094
		Popular	-0.11	-0.32	0.11	0.11	-0.06	-0.99	0.321
Egocentric Bias -PTFC*	491	Literary	-0.21	-0.43	0.02	0.11	-0.12	-1.8	0.073
		Popular	-0.05	-0.28	0.18	0.12	-0.03	-0.45	0.656
Mind Accuracy	493	Literary	5.51	3.54	8.71	1.31	0.27	4.66	< .001
		Popular	-0.28	-3.36	1.95	1.35	-0.01	-0.52	0.601
Social Accuracy*	492	Literary	0.26	0.12	0.41	0.07	0.24	3.53	< .001
		Popular	-0.06	-0.21	0.09	0.08	-0.05	-0.77	0.439

*Variable coding that was not pre-registered.

**Table 2:** Correlations.

		1	2	3	4	5	6
1	Attributional Complexity (N = 493)	-					
2	Egocentric Bias-TFC (N = 491)	*-0*.*08*	*-*				
3	Egocentric Bias -PTFC (N = 491)	-0.07	**0.88**	**-**			
4	Mind Accuracy (N = 493)	**0.26**	-0.04	-0.06	-		
5	Social Accuracy (N = 492)	**0.13**	**0.08**	-0.03	**0.28**	**-**	
6	Education (N = 493)	**0.17**	0	0.01	**0.09**	**0.08**	-

Correlations in bold: p < .05; in italic: p < .10.

### Supplementary analyses

Given the correlational nature of the data we attempted to rule out the effect of potential correlates of exposure to types of fiction. Level of education is a likely candidate, since print exposure and language and comprehension reading skills are moderated by years of education [50] and, in particular, education has been found to be associated with scores on the Mind Accuracy variable (RMET) and the ART [31]. Education correlated with exposure to Literary (*r* = .29, p < .001) and Popular (*r* = .22, p < .001) fiction, with Attributional Complexity (*r* = .17, p < .001), and, marginally, with Mind Accuracy (*r* = .09, p < .07). An ANOVA revealed a main effect of Gender on exposure to both Literary, *F*(1, 487) = 4.93, *p* = .03, and Popular, *F*(1,487) = 14.74, *p* < .001, fiction: females were more familiar than males with both Literary (0.31 vs. 0.26) and Popular (0.31 vs. 0.24) fiction. Females outperformed men on Attributional Complexity (5.35 vs. 5.05), *F*(1,487) = 12.50, *p* < .001, Mind Accuracy (26.69 vs. 25.14), *F*(1,487) = 14.71, *p* < .001, and, marginally, on Social Accuracy (.61 vs. .57), *F*(1,487) = 3.18, *p* < .07. The effect of Gender was not significant for either egocentric bias indicators: TFC (0.02 vs. 0.04), *F*(1,487) = 0.45, *p* = .50; P-TFC (0.28 vs. 0.30), *F*(1,487) = 0.27, *p* = .60.

We thus reran all the analyses reported above by adding education and gender as covariates and excluding two participants who did not report their gender. Fluency in English is important to fully comprehend instructions and can impact participants’ answers to many if not all the variables investigated here. It is important, for instance, to understand the various answer options in the Mind Accuracy measure, or to appreciate the meaning of the items of the Attributional Complexity scale. In the supplementary analyses, therefore, 14 participants (2.87%) who reported being non-native English speakers were excluded.

The new sample was thus 477. With two exceptions, no change in the pattern of relationships and statistical significance indicators emerged. The two exceptions provide stronger support for the egocentric bias hypothesis, since the effect of exposure of Literary fiction emerged as a more reliable predictor of both the TFC, *b* = -0.22, 95% CI = [-0.44, 0.01, *t*(469) = -2.01, *p* = .04, and the P-TFC, *b* = -0.25, 95% CI = [-0.49, -0.02, *t*(469) = -2.13, *p* = .03.

## Discussion

In this article we put forward and tested the hypothesis that engagement with literary fiction, compared to popular fiction, is associated with greater attributional complexity, less egocentric bias, and greater mind accuracy and social accuracy. In support of the first hypothesis, exposure to literary fiction positively predicted attributional complexity, while exposure to popular fiction predicted it negatively. This finding is important for several reasons. First, it contributes to the ongoing discourse surrounding the role of fiction in shaping social cognition (e.g., [7]) and is consistent with theory and research on the characteristics of literary fiction and how they may impact cognition and cognitive style [25, 30]. Second, understanding correlates and especially possible predictors of attributional complexity is of importance and has far ranging potential consequences because high attributional complexity attenuates racism [51], and plays a role in attitudes about important policy-related opinions [52].

Behavioral measures of attributional complexity have also been found to correlate with mind-reading accuracy [53] and to predict accuracy in attribution made by managers in work contexts [54]. The pattern of correlations revealed in this study is consistent with both of these findings, since we found that attributional complexity correlated positively with both mind accuracy and social accuracy. More broadly, the rationale regarding attributional complexity put forward in this paper, and the pattern we observed, is consistent with previous work showing that those who score high in attributional complexity are described by peers as possessing social wisdom and thoughtfulness [55].

The second hypothesis, that exposure to literary fiction is associated with less egocentric bias, received only marginal support in the pre-registered analyses. Stronger support emerged after adding control variables and excluding non-native English speakers. Contrary to the rest, these supplementary analyses were not preregistered, so these results should be interpreted with caution.

The third hypothesis was supported: Exposure to literary fiction predicted performance on both accuracy measures. The mind accuracy result replicates previous findings [25, 31] and is consistent with findings from experimental studies that manipulated, instead of measured, exposure to literary and popular fiction (e.g., [25, 30, 34, 35, 56]). The result observed on the social accuracy measure is novel, suggesting that exposure to literary fiction may be associated with better social skills not only when it comes to interpreting the thoughts and emotions of another individual’s mind, but also with a more accurate reading of the social landscape.

Consistent with our rationale, and as implied by our use of regression analyses, is the idea that exposure to fiction influences socio-cognitive processes and styles. Yet, even though this rationale is consistent with previous work that is experimental in nature, because of the correlational nature of the data presented here, we caution against drawing strong conclusions about causality. Our view is that reading different kinds of fiction fosters certain socio-cognitive processes and cognitive styles, relative to others, but we also agree that individual differences in these processes and styles may make people more prone to gravitate toward different kinds of fiction.

### The ART as a proxy for fiction reading

To assess the impact of exposure to fiction we used the Author Recognition Test. As discussed above, the ART is not a direct measure of fiction reading, however previous research has concluded that the ART is a good measure of actual exposure to fiction and is less likely to suffer from social desirability concerns, compared to other self-report measures [39–41].

Notwithstanding the qualities of the ART, it can be argued that recognition of authors may be the consequence of exposure to their names in contexts that have little to do with actual fiction reading. While it cannot be firmly excluded that third variables may be responsible for the correlation between ART and socio-cognitive variables, research studies have successfully excluded the confounding role of a series of factors such as age, gender, experience with the English language, intelligence, transportation (in the narrative) tendency, and personality traits [42, 43]. Some of these potentially confounding factors were also tested and controlled for in the present data.

In this article, however, we did not only use the ART to measure exposure to fiction in general. Instead, we computed two scores tapping exposure to literary and popular fiction. When the ART is used this way, other confounding variables may also need to be considered. Given the stereotypes about audiences for literary and popular fiction, one may argue that Socio Economic Status (SES) may be one such variable. We did not measure SES in our study, and are aware of no studies that have looked at the relationship between ART and SES. We did, however, measure level of education, which correlates positively with SES [57, 58], and we found that covarying education level did not alter our pattern of findings. Furthermore, SES is negatively, not positively associated with at least one of the socio-cognitive variables we measured, namely our measure of mind accuracy, the RMET [59, 60].

Beside overall level of education, a further factor that may impact individuals’ exposure to fiction is college major, with those majoring in the humanities being more likely to be exposed to literary fiction, compared to other majors. Research shows that undergraduate major impacts ART scores on both literary and popular fiction, and while the effect size of literary fiction is larger than that on popular fiction, controlling for major does not change the differential effect of literary and popular fiction on the RMET [31]. Furthermore, it should also be noted that, if anything, exposure to literary fiction would be the mediating factor between university major and socio-cognitive skills. Discussing the factors that influence exposure to literary fiction is not the goal of this article, but we would certainly agree that college major may be one of them.

All in all, while the ART has limitations, it is a valuable measure of exposure to fiction that has built-in controls for social desirability responding, and can also be used to differentiate between exposure to literary and popular fiction [46] in order to assess how they differentially predict social cognition processes and cognitive style [31]. Yet, further research is certainly needed. As mentioned above, experimental evidence already exists demonstrating that compared to popular, literary fiction enhances Theory of Mind (e.g., [30]). To our knowledge, however, the results presented here are the first evidence that literary fiction uniquely enhances attributional complexity and the estimation of social attitudes (social accuracy). Although the present results are not as clear in this regard, exposure to literary fiction may also diminish ego-centric bias.

Further research, especially studies that manipulate rather than measure exposure to these two types of fiction, and assess their effect on these other variables, are needed. These studies may be conducted in more controlled environments, compared to the online environment in which we conducted the present studies. This being said, the present data was collected via MTurk, which has proven a valid source for behavioral research [37]. The fact that our hypotheses were pre-registered also strengthens our confidence in the findings.

### Is literary fiction better than popular fiction?

The rationale presented above, according to which literary and popular fiction may prompt different social cognition processes and promote different cognitive styles, as well as the pattern of findings emerging from this study, should not be interpreted as a suggestion that literary fiction is *better* than popular fiction.

For one thing, the variables that are specifically associated with exposure to literary fiction may be desirable from one perspective, but problematic from other perspectives. Literary fiction is associated with greater attributional complexity, which seems a valuable cognitive style from a societal perspective. Yet, attributional complexity may also delay or derail decision-making and it has been shown to be negatively related to mental health [61]. Literary fiction is, somewhat, associated with lower ego-centric bias, but it should be noted that ego-centric bias is positively related to mental health [62]. Other possible negative consequences of exposure to literary fiction include the fact that an increase in social accuracy may be nefarious for interpersonal relations [63].

We also speculate that literary and popular fiction may have opposite effects on anxiety. According to Terror Management Theory [64], different forms of anxieties are instantiations of the fundamental existential anxiety that all humans experience as a consequence of their knowledge of the inevitability of death [65]. One of the most important psychological mechanisms through which we keep this existential anxiety at bay is cultural worldviews: conceptions of reality that imbue life with stability, order, and permanence. Cultural worldviews are elaborated and maintained within cultural ingroups [66] through a variety of cultural artefacts, among which is fiction. Given the characteristics of literary and popular fiction discussed above, we would predict that exposure to popular fiction (because it confirms expectations about the world) reduces existential anxiety, while literary fiction (because it challenges such expectations) enhances it.

All in all, while some cognitive processes and styles associated with reading literary fiction are considered desirable from a social psychological standpoint (e.g. reducing prejudicial attitudes) they may be undesirable from a health psychology perspective (e.g. compromising mental health). There is, however, another reason why we reject a hierarchy of fiction. Literary and popular fiction foster different socio-cognitive processes and cognitive styles, all of which are important.

### Binding and individuating

Some of the socio-cognitive processes and cognitive styles discussed above facilitate the binding among humans that is functional to forming and maintaining social groups. These binding processes allow us to imagine [67], constitute and maintain social groups, notably through the process of self and social categorization [68] and the development of social identities [69], as well as through the identification and the enacting of social roles [70]. Other processes and styles do the opposite; they are individuating and facilitate and thus also foster a view of the world in terms of unique individuals, as opposed to social groups. This distinction has many parallels in social psychological theory (e.g., [71, 72]), but a discussion of this work goes well beyond the scope of this article. Here we mention it simply to stress that we should look at literary and popular fiction as fostering individuating *versus* binding, respectively. From this perspective, a hierarchy of fiction is meaningless because both binding and individuating is needed not only at the societal level for human societies to function and evolve (e.g., [72]) but also, when directed inward, to satisfy intra-psychic needs [71].

We *homo sapiens* “made it” while the Neanderthals did not, because we developed group coordination, and hunted and defended ourselves not as individuals, but as a community [73, 74]. We do not, however, operate like a swarm of honeybees or a line of worker ants; our existence is not subsumed into that of our society. We are unique individuals, with somewhat idiosyncratic characteristics and a sense of individual self. While, as put by John Donne, “No man is an island,” we are also a society of the selves [23].

A thriving human society must therefore allow, and in fact maintain, a continuous tension between these two seemingly incompatible sets of processes—those that are individuating and those that are binding. It is thus reasonable that the tension between these two psychological and societal forces can be found in many domains of human life, including the arts, between tradition and avant-garde, and we contend, in fiction, between popular and literary.
